# Supplementary material for: Experimental and Computational Studies on Structure and Energetic Properties of Halogen Derivatives of 2-Deoxy-D-Glucose
Source: Int J Mol Sci. 2021 Apr 2;22(7):3720. doi: 10.3390/ijms22073720 (PMC8038202; doi:10.3390/ijms22073720)
Supplement: Supplementary file 1 [file ijms-22-03720-s001.pdf]

International Journal of
Molecular Sciences

MDPI

Supplementary materials

Table S1. Crystal data and structure refinement details for investigated compounds.

Compound	2-FG	2-CG	2-IG	2-FM	2-CM
Empirical formula	$C_6H_{11}FO_5$	$C_6H_{11}ClO_5$	$C_6H_{11}IO_5$	$C_6H_{11}FO_5$	$C_6H_{13}ClO_6$
Formula weight	182.15	198.60	290.05	182.15	216.61
Temperature/K	100(2)	100(2)	100(2)	100(2)	100(2)
Crystal system	orthorhombic	orthorhombic	orthorhombic	orthorhombic	monoclinic
Space group	$P2_12_12_1$	$P2_12_12_1$	$P2_12_12_1$	$P2_12_12_1$	$P2_1$
a/Å	6.4109(5)	6.7410(3)	6.3515(3)	6.5318(6)	6.1125(2)
b/Å	10.4044(6)	6.8408(2)	6.6674(3)	10.3558(4)	7.3669(2)
c/Å	11.2128(8)	17.1401(6)	19.9328(8)	11.1207(7)	10.1426(4)
α/°	90	90	90	90	90
β/°	90	90	90	90	106.020(4)
γ/°	90	90	90	90	90
Volume/Å^3	747.91(9)	790.40(5)	844.12(6)	752.22(8)	438.99(3)
Z	4	4	4	4	2
ρ_{calc}g/cm^3	1.618	1.669	2.282	1.608	1.639
μ/mm^{-1}	1.372	4.204	29.743	1.364	3.923
F(000)	384.0	416.0	560.0	384.0	228.0
Crystal size/mm^3	0.22 × 0.06 × 0.06	0.12 × 0.05 × 0.04	0.08 × 0.05 × 0.04	0.13 × 0.11 × 0.03	0.27 × 0.13 × 0.06
Radiation	Cu$K\alpha$ (λ = 1.54184)	CuKα (λ = 1.54184)	Cu$K\alpha$ (λ = 1.54184)	Cu$K\alpha$ (λ = 1.54184)	Cu$K\alpha$ (λ = 1.54184)
2Θ range for data collection/°	11.602 to 134.152	10.322 to 134.108	8.872 to 134.134	11.676 to 134.148	9.072 to 134.082
Index ranges	$-7 \le h \le 7$, $-12 \le k \le 12$, $-13 \le l \le 13$	$-8 \le h \le 8$, $-8 \le k \le 8$, $-20 \le l \le 20$	$-7 \le h \le 7$, $-7 \le k \le 7$, $-23 \le l \le 23$	$-7 \le h \le 7$, $-12 \le k \le 12$, $-13 \le l \le 13$	$-7 \le h \le 7$, $-8 \le k \le 8$, $-12 \le l \le 12$
Reflections collected	10763	10825	11303	11274	5555
Independent reflections	1341 [R_{int} = 0.0775, R_{sigma} = 0.0362]	1410 [R_{int} = 0.0431, R_{sigma} = 0.0215]	1498 [R_{int} = 0.0463, R_{sigma} = 0.0233]	1343 [R_{int} = 0.0585, R_{sigma} = 0.0307]	1559 [R_{int} = 0.0181, R_{sigma} = 0.0144]
Data/restraints/parameters	1341/4/121	1410/4/121	1498/4/121	1343/4/121	1559/8/138
Goodness-of-fit on F^2	1.081	1.071	1.105	1.067	1.112
Final R indexes [I>=2σ (I)]	R_1 = 0.0417, wR_2 = 0.1068	R_1 = 0.0245, wR_2 = 0.0587	R_1 = 0.0290, wR_2 = 0.0700	R_1 = 0.0476, wR_2 = 0.1244	R_1 = 0.0193, wR_2 = 0.0515
Final R indexes [all data]	R_1 = 0.0447, wR_2 = 0.1096	R_1 = 0.0270, wR_2 = 0.0602	R_1 = 0.0291, wR_2 = 0.0700	R_1 = 0.0528, wR$_2$ = 0.1285	R_1 = 0.0195, wR_2 = 0.0519
Largest diff. peak/hole / e Å^{-3}	0.36/-0.28	0.25/-0.19	1.26/-1.00	0.63/-0.25	0.21/-0.17
Flack parameter	0.08(14)	-0.013(8)	-0.017(9)	0.01(17)	-0.019(10)

Table S2. Bond lengths for 2-FG

Atom	Atom	Length/Å	Atom	Atom	Length/Å
C(1)	C(2)	1.521(5)	C(3)	O(3)	1.422(4)
C(1)	O(1)	1.387(4)	C(4)	C(5)	1.522(4)
C(1)	O(5)	1.432(4)	C(4)	O(4)	1.428(4)
C(2)	C(3)	1.520(4)	C(5)	C(6)	1.516(4)
C(2)	F(1)	1.408(4)	C(5)	O(5)	1.438(4)
C(3)	C(4)	1.528(5)	C(6)	O(6)	1.432(4)

Table S3. Values of valence angles for 2-FG

Atom	Atom	Atom	Angle/°	Atom	Atom	Atom	Angle/°
O(1)	C(1)	C(2)	109.0(3)	C(5)	C(4)	C(3)	111.1(3)
O(1)	C(1)	O(5)	108.2(2)	O(4)	C(4)	C(3)	111.4(2)
O(5)	C(1)	C(2)	108.6(2)	O(4)	C(4)	C(5)	106.9(2)
C(3)	C(2)	C(1)	112.3(3)	C(6)	C(5)	C(4)	113.8(3)
F(1)	C(2)	C(1)	108.0(3)	O(5)	C(5)	C(4)	109.0(2)
F(1)	C(2)	C(3)	108.2(2)	O(5)	C(5)	C(6)	107.4(2)
C(2)	C(3)	C(4)	110.8(2)	O(6)	C(6)	C(5)	109.3(3)
O(3)	C(3)	C(2)	109.6(3)	C(1)	O(5)	C(5)	112.5(2)
O(3)	C(3)	C(4)	109.0(3)				

Table S4. Values of torsion angles for 2-FG

A	B	C	D	Angle/°	A	B	C	D	Angle/°
C(1)	C(2)	C(3)	C(4)	-49.1(3)	F(1)	C(2)	C(3)	O(3)	71.3(3)
C(1)	C(2)	C(3)	O(3)	-169.5(2)	O(1)	C(1)	C(2)	C(3)	172.8(3)
C(2)	C(1)	O(5)	C(5)	-63.9(3)	O(1)	C(1)	C(2)	F(1)	-67.9(3)
C(2)	C(3)	C(4)	C(5)	49.2(3)	O(1)	C(1)	O(5)	C(5)	177.9(2)
C(2)	C(3)	C(4)	O(4)	168.3(3)	O(3)	C(3)	C(4)	C(5)	169.9(2)
C(3)	C(4)	C(5)	C(6)	-175.8(2)	O(3)	C(3)	C(4)	O(4)	-71.0(3)
C(3)	C(4)	C(5)	O(5)	-55.9(3)	O(4)	C(4)	C(5)	C(6)	62.5(3)
C(4)	C(5)	C(6)	O(6)	59.7(3)	O(4)	C(4)	C(5)	O(5)	-177.7(2)
C(4)	C(5)	O(5)	C(1)	64.8(3)	O(5)	C(1)	C(2)	C(3)	55.2(3)
C(6)	C(5)	O(5)	C(1)	-171.4(3)	O(5)	C(1)	C(2)	F(1)	174.4(2)
F(1)	C(2)	C(3)	C(4)	-168.3(3)	O(5)	C(5)	C(6)	O(6)	-61.0(3)

Table S5. Bond lengths for 2-DG

Atom	Atom	Length/Å	Atom	Atom	Length/Å
C(1)	C(2)	1.536(3)	C(3)	O(3)	1.425(3)
C(1)	O(1)	1.387(3)	C(4)	C(5)	1.522(4)
C(1)	O(5)	1.427(3)	C(4)	O(4)	1.428(3)
C(2)	C(3)	1.522(3)	C(5)	C(6)	1.510(4)
C(2)	Cl(1)	1.807(2)	C(5)	O(5)	1.437(3)
C(3)	C(4)	1.526(4)	C(6)	O(6)	1.426(3)

Table S6. Values of valence angles for 2-CG

Atom	Atom	Atom	Angle/°	Atom	Atom	Atom	Angle/°
O(1)	C(1)	C(2)	113.1(2)	C(5)	C(4)	C(3)	110.0(2)
O(1)	C(1)	O(5)	108.4(2)	O(4)	C(4)	C(3)	109.9(2)
O(5)	C(1)	C(2)	108.10(19)	O(4)	C(4)	C(5)	107.88(19)
C(1)	C(2)	Cl(1)	108.70(16)	C(6)	C(5)	C(4)	114.0(2)
C(3)	C(2)	C(1)	111.4(2)	O(5)	C(5)	C(4)	107.9(2)
C(3)	C(2)	Cl(1)	109.22(18)	O(5)	C(5)	C(6)	109.3(2)
C(2)	C(3)	C(4)	110.3(2)	O(6)	C(6)	C(5)	115.0(2)
O(3)	C(3)	C(2)	108.3(2)	C(1)	O(5)	C(5)	111.0(2)
O(3)	C(3)	C(4)	110.4(2)				

Table S7. Values of torsion angles for 2-CG

A	B	C	D	Angle/°	A	B	C	D	Angle/°
C(1)	C(2)	C(3)	C(4)	-50.0(3)	Cl(1)	C(2)	C(3)	O(3)	69.0(2)
C(1)	C(2)	C(3)	O(3)	-170.93(19)	O(1)	C(1)	C(2)	C(3)	176.0(2)
C(2)	C(1)	O(5)	C(5)	-65.9(2)	O(1)	C(1)	C(2)	Cl(1)	-63.5(2)
C(2)	C(3)	C(4)	C(5)	51.7(3)	O(1)	C(1)	O(5)	C(5)	171.16(19)
C(2)	C(3)	C(4)	O(4)	170.3(2)	O(3)	C(3)	C(4)	C(5)	171.3(2)
C(3)	C(4)	C(5)	C(6)	178.8(2)	O(3)	C(3)	C(4)	O(4)	-70.0(2)
C(3)	C(4)	C(5)	O(5)	-59.6(3)	O(4)	C(4)	C(5)	C(6)	58.9(3)
C(4)	C(5)	C(6)	O(6)	56.6(3)	O(4)	C(4)	C(5)	O(5)	-179.52(19)
C(4)	C(5)	O(5)	C(1)	68.4(3)	O(5)	C(1)	C(2)	C(3)	56.0(3)
C(6)	C(5)	O(5)	C(1)	-167.1(2)	O(5)	C(1)	C(2)	Cl(1)	176.45(16)
Cl(1)	C(2)	C(3)	C(4)	-170.15(16)	O(5)	C(5)	C(6)	O(6)	-64.3(3)

Table S8. Bond lengths for 2-IG

Atom	Atom	Length/Å	Atom	Atom	Length/Å
C(1)	C(2)	1.528(11)	C(3)	O(2)	1.426(10)
C(1)	O(1)	1.352(12)	C(4)	C(5)	1.534(11)
C(1)	O(5)	1.429(10)	C(4)	O(3)	1.422(10)
C(2)	C(3)	1.508(12)	C(5)	C(6)	1.510(12)
C(2)	I(1)	2.167(8)	C(5)	O(5)	1.460(10)
C(3)	C(4)	1.518(12)	C(6)	O(6)	1.421(10)

Table S9. Values of valence angles for 2-IG

Atom	Atom	Atom	Angle/°	Atom	Atom	Atom	Angle/°
O(1)	C(1)	C(2)	111.8(7)	C(3)	C(4)	C(5)	109.4(7)
O(1)	C(1)	O(5)	112.5(7)	O(3)	C(4)	C(3)	110.6(7)
O(5)	C(1)	C(2)	107.6(7)	O(3)	C(4)	C(5)	109.3(7)
C(1)	C(2)	I(1)	109.3(5)	C(6)	C(5)	C(4)	115.0(7)
C(3)	C(2)	C(1)	114.0(7)	O(5)	C(5)	C(4)	107.5(7)
C(3)	C(2)	I(1)	110.9(5)	O(5)	C(5)	C(6)	108.4(7)
C(2)	C(3)	C(4)	108.2(7)	O(6)	C(6)	C(5)	113.7(7)
O(2)	C(3)	C(2)	110.5(7)	C(1)	O(5)	C(5)	113.9(6)
O(2)	C(3)	C(4)	108.1(7)				

Table S10. Values of torsion angles for 2-IG

A	B	C	D	Angle/°	A	B	C	D	Angle/°
C(1)	C(2)	C(3)	C(4)	-54.2(9)	I(1)	C(2)	C(3)	O(2)	63.9(8)
C(1)	C(2)	C(3)	O(2)	-172.3(7)	O(1)	C(1)	C(2)	C(3)	-69.8(9)
C(2)	C(1)	O(5)	C(5)	-59.5(9)	O(1)	C(1)	C(2)	I(1)	54.9(8)
C(2)	C(3)	C(4)	C(5)	56.5(9)	O(1)	C(1)	O(5)	C(5)	64.2(9)
C(2)	C(3)	C(4)	O(3)	177.0(7)	O(2)	C(3)	C(4)	C(5)	176.1(7)
C(3)	C(4)	C(5)	C(6)	178.2(7)	O(2)	C(3)	C(4)	O(3)	-63.4(9)
C(3)	C(4)	C(5)	O(5)	-60.9(8)	O(3)	C(4)	C(5)	C(6)	56.9(9)
C(4)	C(5)	C(6)	O(6)	51.1(10)	O(3)	C(4)	C(5)	O(5)	177.8(6)
C(4)	C(5)	O(5)	C(1)	64.4(8)	O(5)	C(1)	C(2)	C(3)	54.2(9)
C(6)	C(5)	O(5)	C(1)	-170.7(6)	O(5)	C(1)	C(2)	I(1)	178.9(5)
I(1)	C(2)	C(3)	C(4)	-178.0(5)	O(5)	C(5)	C(6)	O(6)	-69.2(8)

Table S11. Bond lengths for 2-FM

Atom	Atom	Length/Å	Atom	Atom	Length/Å
C(1)	C(2)	1.514(6)	C(3)	O(3)	1.421(5)
C(1)	O(1)	1.386(5)	C(4)	C(5)	1.524(5)
C(1)	O(5)	1.435(5)	C(4)	O(4)	1.426(5)
C(2)	C(3)	1.522(6)	C(5)	C(6)	1.510(6)
C(2)	F(1)	1.415(4)	C(5)	O(5)	1.441(5)
C(3)	C(4)	1.523(6)	C(6)	O(6)	1.431(5)

Table S12. Values of valence angles for 2-FM

Atom	Atom	Atom	Angle/°	Atom	Atom	Atom	Angle/°
O(1)	C(1)	C(2)	109.1(3)	C(3)	C(4)	C(5)	110.5(4)
O(1)	C(1)	O(5)	107.4(3)	O(4)	C(4)	C(3)	112.7(3)
O(5)	C(1)	C(2)	109.9(3)	O(4)	C(4)	C(5)	107.5(3)
C(1)	C(2)	C(3)	112.2(3)	C(6)	C(5)	C(4)	113.4(4)
F(1)	C(2)	C(1)	107.6(3)	O(5)	C(5)	C(4)	109.1(3)
F(1)	C(2)	C(3)	107.7(3)	O(5)	C(5)	C(6)	107.3(3)
C(2)	C(3)	C(4)	110.2(3)	O(6)	C(6)	C(5)	109.2(3)
O(3)	C(3)	C(2)	109.6(3)	C(1)	O(5)	C(5)	112.8(3)
O(3)	C(3)	C(4)	109.8(4)				

Table S13. Values of torsion angles for 2-FM

A	B	C	D	Angle/°	A	B	C	D	Angle/°
C(1)	C(2)	C(3)	C(4)	-50.6(4)	F(1)	C(2)	C(3)	O(3)	-53.3(4)
C(1)	C(2)	C(3)	O(3)	-171.5(3)	O(1)	C(1)	C(2)	C(3)	171.4(3)
C(2)	C(1)	O(5)	C(5)	-60.9(4)	O(1)	C(1)	C(2)	F(1)	53.1(4)
C(2)	C(3)	C(4)	C(5)	52.3(4)	O(1)	C(1)	O(5)	C(5)	-179.5(3)
C(2)	C(3)	C(4)	O(4)	172.5(3)	O(3)	C(3)	C(4)	C(5)	173.1(3)
C(3)	C(4)	C(5)	C(6)	-177.3(3)	O(3)	C(3)	C(4)	O(4)	-66.7(4)
C(3)	C(4)	C(5)	O(5)	-57.8(4)	O(4)	C(4)	C(5)	C(6)	59.4(4)
C(4)	C(5)	C(6)	O(6)	56.4(4)	O(4)	C(4)	C(5)	O(5)	178.9(3)
C(4)	C(5)	O(5)	C(1)	63.1(4)	O(5)	C(1)	C(2)	C(3)	53.9(4)
C(6)	C(5)	O(5)	C(1)	-173.6(3)	O(5)	C(1)	C(2)	F(1)	-64.4(4)
F(1)	C(2)	C(3)	C(4)	67.6(4)	O(5)	C(5)	C(6)	O(6)	-64.2(4)

Table S14. Bond lengths for 2-CM

Atom	Atom	Length/Å	Atom	Atom	Length/Å
C(1)	C(2)	1.526(3)	C(3)	O(3)	1.425(3)
C(1)	O(1)	1.381(3)	C(4)	C(5)	1.530(3)
C(1)	O(5)	1.436(3)	C(4)	O(4)	1.425(3)
C(2)	C(3)	1.531(3)	C(5)	C(6)	1.511(3)
C(2)	Cl(1)	1.796(2)	C(5)	O(5)	1.442(3)
C(3)	C(4)	1.524(3)	C(6)	O(6)	1.424(3)

Table S15. Values of valence angles for 2-CM

Atom	Atom	Atom	Angle/°	Atom	Atom	Atom	Angle/°
O(1)	C(1)	C(2)	111.11(18)	C(3)	C(4)	C(5)	110.02(16)
O(1)	C(1)	O(5)	107.85(17)	O(4)	C(4)	C(3)	107.27(16)
O(5)	C(1)	C(2)	110.07(17)	O(4)	C(4)	C(5)	110.13(18)
C(1)	C(2)	C(3)	110.26(18)	C(6)	C(5)	C(4)	111.09(16)
C(1)	C(2)	Cl(1)	109.99(15)	O(5)	C(5)	C(4)	109.3(2)
C(3)	C(2)	Cl(1)	110.44(14)	O(5)	C(5)	C(6)	107.99(16)
C(4)	C(3)	C(2)	111.29(16)	O(6)	C(6)	C(5)	111.94(17)
O(3)	C(3)	C(2)	110.41(18)	C(1)	O(5)	C(5)	111.37(15)
O(3)	C(3)	C(4)	110.51(17)				

Table S16. Values of torsion angles for 2-CM

A	B	C	D	Angle/°	A	B	C	D	Angle/°
C(1)	C(2)	C(3)	C(4)	-50.7(2)	Cl(1)	C(2)	C(3)	O(3)	-52.1(2)
C(1)	C(2)	C(3)	O(3)	-173.85(17)	O(1)	C(1)	C(2)	C(3)	175.23(17)
C(2)	C(1)	O(5)	C(5)	-63.9(2)	O(1)	C(1)	C(2)	Cl(1)	53.2(2)
C(2)	C(3)	C(4)	C(5)	51.6(2)	O(1)	C(1)	O(5)	C(5)	174.76(17)
C(2)	C(3)	C(4)	O(4)	171.43(17)	O(3)	C(3)	C(4)	C(5)	174.69(18)
C(3)	C(4)	C(5)	C(6)	-176.57(18)	O(3)	C(3)	C(4)	O(4)	-65.5(2)
C(3)	C(4)	C(5)	O(5)	-57.5(2)	O(4)	C(4)	C(5)	C(6)	65.4(2)
C(4)	C(5)	C(6)	O(6)	-174.60(18)	O(4)	C(4)	C(5)	O(5)	-175.52(16)
C(4)	C(5)	O(5)	C(1)	64.5(2)	O(5)	C(1)	C(2)	C(3)	55.8(2)
C(6)	C(5)	O(5)	C(1)	-174.46(17)	O(5)	C(1)	C(2)	Cl(1)	-66.2(2)
Cl(1)	C(2)	C(3)	C(4)	71.0(2)	O(5)	C(5)	C(6)	O(6)	65.5(2)

Table S17. Cremer-Pople parameters for crystal structures

Compound	q_2	q_3	Q	θ [°]	φ_2 [°]
2-DG (α anomer)	0.060	0.548	0.551	6.0	256.0
2-DG (β anomer)	0.038	0.562	0.563	3.9	350.4
2-FG	0.075	0.563	0.568	7.1	355.0
2-CG	0.085	0.590	0.596	7.8	344.2
2-IG	0.057	0.588	0.591	5.5	288.0
2-FM	0.051	0.565	0.567	5.2	327.0
2-CM	0.061	0.577	0.581	6.0	348.0

Table S18. The geometry of hydrogen bonds in the crystal of *α*-2-DG

D–H	A	D–H (Å)	d(D···A) (Å)	< D–H···A (°)
O1–H1	O4^{i}	0.84	2.780(2)	171
O2–H2	O1^{ii}	0.84	2.784(2)	155
O3–H3	O5^{iii}	0.84	2.766(2)	174
O5–H4	O2^{iv}	0.84	2.670(2)	170
C2–H6	O3^{v}	0.99	3.390(3)	148
C2–H7	O1^{vi}	0.99	3.344(3)	142

Symmetry codes: (i) *x* + 1/2, –*y* + 1/2, –*z* + 1; (ii) –*x* + 1, *y* + 1/2, –*z* + 1/2; (iii) *x* – 1/2, –*y* + 3/2, –*z* + 1; (iv) –*x* + 1/2, –*y* + 1, *z* + 1/2; (v) –*x* +1, *y* – 1/2, –*z* + 1/2; (vi) *x* – 1, *y*, *z*.

Table S19. The geometry of hydrogen bonds in the crystal of *b*-2-DG

D–H	A	D–H (Å)	d(D···A) (Å)	< D–H···A (°)
O1–H9	O5^{i}	0.94	2.655(2)	169
O2–H10	O4^{ii}	0.61	2.739(2)	172
O3–H11	O1^{ii}	0.76	2.808(2)	150
O5–H12	O2^{iii}	0.76	2.697(2)	165
C5–H6	O3^{iv}	1.07	3.380(2)	147
C6–H8	O1^{v}	1.08	3.518(2)	146

Symmetry codes: (i) *x* − 1, *y*, *z*; (ii) −*x* + 1, *y* − 1/2, −*z* + 1/2; (iii) −*x* + 3/2, −*y* + 1, *z* + 1/2; (iv) *x* − 1/2, −*y* + 1/2, −*z* + 1; (v) −*x* + 1/2, −*y* + 1, *z* + 1/2.

Table S20. The geometry of hydrogen bonds in the crystal of 2-FG

D–H	A	D–H (Å)	d(D···A) (Å)	< D–H···A (°)
O1–H1A	O6^{i}	0.83(4)	2.622(3)	172(5)
O3–H3A	O5^{ii}	0.83(2)	2.785(3)	166(4)
O4–H4A	O3*	0.83(4)	2.955(3)	110(3)
O4–H4A	O1^{ii}	0.83(4)	2.786(3)	149(4)
O6–H6	O3^{iii}	0.83(4)	2.714(3)	175(4)
C5–H5	O4^{iv}	0.98	3.353(4)	147
C6–H6A	F1^{v}	0.97	3.325(4)	142

Symmetry codes: (i) *x* − 1, *y* ,*z*; (ii) −*x* + 1, *y* − 1/2, −*z* + 1/2; (iii) −*x* + 3/2, −*y* + 1, *z* + 1/2; (iv) *x* − 1/2, −*y* + 1/2, −*z* + 1; (v) −*x* + 1/2, −*y* + 1, *z* + 1/2; (*) intramolecular interaction.

Table S21. The geometry of hydrogen bonds in the crystal of 2-CG

D–H	A	D–H (Å)	d(D···A) (Å)	< D–H···A (°)
O1–H1A	O3^{i}	0.82(2)	2.683(3)	164(3)
O3–H3A	O6^{ii}	0.81(3)	2.679(3)	172(3)
O4–H4A	Cl^{iii}	0.82(3)	3.321(2)	167(2)
O6–H6	O1^{iii}	0.82(3)	2.771(3)	171(3)
C2–H2	O3^{i}	0.98	3.237(3)	128
C5–H5	O4^{iv}	0.98	3.485(3)	157

Symmetry codes: (i) *x* + 1/2, –*y* + 1/2, –*z* + 1; (ii) *x* – 1, *y*, *z*; (iii) *x*, *y* + 1, *z*; (iv) –*x* + 1, *y* – 1/2, –*z* + 1/2.

Table S22. The geometry of hydrogen bonds in the crystal of 2-IG

D–H	A	D–H (Å)	d(D···A) (Å)	< D–H···A (°)
O1–H1A	O6^{i}	0.82(7)	2.648(9)	151(11)
O2–H2A	O5^{ii}	0.82(8)	2.771(8)	167(11)

O3–H3A	O1^{iii}	0.82(9)	2.951(10)	153(13)
O6–H6	O2^{iv}	0.82(5)	2.832(9)	127(8)
O6–H6	O3^{iv}	0.82(5)	3.169(9)	140(9)

Symmetry codes: (i) *x*, *y* − 1, *z*; (ii) *x* − 1, *y*, *z*; (iii) *x*, *y* + 1, *z*; (iv) *x* + 1, *y*, *z*.

Table S23. The geometry of hydrogen bonds in the crystal of 2-FM

D–H	A	D–H (Å)	d(D···A) (Å)	< D–H···A (°)
O1–H1A	O6^{i}	0.83(5)	2.665(4)	165(6)
O3–H3A	O5^{ii}	0.82(2)	2.726(3)	171(6)
O4–H4A	O3*	0.82(4)	2.940(4)	107(4)
O4–H4A	O1^{ii}	0.82(4)	2.846(5)	163(5)
O6–H6	F1^{iii}	0.81(4)	3.002(4)	142(5)
O6–H6	O3^{iii}	0.81(4)	2.714(4)	137(4)
C5–H5	O4^{iv}	0.98	3.464(5)	150

Table S24. The geometry of hydrogen bonds in the crystal of 2-CM

D–H	A	D–H (Å)	d(D···A) (Å)	< D–H···A (°)
O1W–H1WA	O1^{i}	0.85(2)	2.851(2)	171(3)
O1W–H1WA	O1W^{ii}	0.82(3)	2.629(2)	165(3)
O1W–H1WB	O6^{iii}	0.86(2)	2.727(2)	175.3(19)
O3–H3A	O5^{i}	0.82(3)	2.814(2)	167(3)
O4–H4A	O3^{iv}	0.81(2)	2.815(2)	161(4)
O6–H6	O4^{v}	0.82(3)	2.663(3)	168(3)
C4–H4	O6^{vi}	0.98	3.496(3)	157

Symmetry codes: (i) *x* − 1, *y*, *z*; (ii) −*x* + 3, *y* − 1/2, −*z* + 1; (iii) *x* − 1, *y* + 1, *z*; (iv) −*x* + 2, *y* − 1/2, −*z*; (v) *x* + 1, *y*, *z*; (vi) −*x* + 3, *y* + 1/2, −*z*.

Table S25. Interaction energies (kJ mol^{-1}) for adjacent molecules

Compound	N	Symmetry operation	R	E_{ele}	E_{pol}	E_{dis}	E_{rep}	E_{tot}
	2	-x, y+1/2, -z+1/2	6.79	-34.1	-6.4	-15.9	45.0	-26.9
	2	x+1/2, -y+1/2, -z	5.95	-47.0	-9.9	-21.4	51.1	-44.1
α-2-DG	2	x, y, z	4.85	-7.8	-2.2	-21.2	13.9	-19.7
	2	-x+1/2, -y, z+1/2	7.93	-52.6	-11.1	-11.1	66.6	-32.4
	2	x+1/2, -y+1/2, -z	6.48	-45.1	-11.2	-22.1	56.9	-40.1
	2	x, y, z	6.48	-56.1	-13.0	-20.1	78.7	-37.8
	2	-x+1/2, -y, z+1/2	6.76	-6.8	-1.6	-11.5	7.5	-13.7
	2	-x, y+1/2, -z+1/2	8.48	-0.2	-0.5	-2.7	0.1	-2.8
β-2-DG	2	x+1/2, -y+1/2, -z	6.01	-2.0	-1.8	-15.0	12.5	-8.8
	2	-x, y+1/2, -z+1/2	5.43	-73.8	-17.5	-27.5	87.6	-60.7
	2	x+1/2, -y+1/2, -z	8.52	-4.5	-0.5	-3.4	0.6	-7.8
	2	-x+1/2, -y, z+1/2	6.73	-41.2	-8.4	-15.5	60.9	-25.7
	2	-x, y+1/2, -z+1/2	5.38	-70.7	-16.6	-28.7	80.0	-62.6
	2	x, y, z	6.41	-64.8	-15.2	-22.5	88.8	-44.4
2-FG	2	-x+1/2, -y, z+1/2	7.06	-40.1	-7.9	-14.4	58.8	-24.4
	2	x+1/2, -y+1/2, -z	6.18	-3.2	-1.9	-15.9	13.8	-10.2
	2	-x+1/2, -y, z+1/2	6.59	-6.2	-1.9	-14.1	11.0	-13.4

	2	x+1/2, -y+1/2, -z	8.19	-2.7	-0.8	-4.5	1.1	-6.7
2-FM	2	-x, y+1/2, -z+1/2	8.46	-1.0	-0.6	-1.8	0.0	-3.1
	2	-x+1/2, -y, z+1/2	6.71	-31.7	-6.5	-18.7	46.3	-26.0
	2	x, y, z	6.53	-48.0	-13.8	-20.8	78.2	-30.8
	2	x+1/2, -y+1/2, -z	6.41	-0.8	-1.8	-14.4	10.2	-8.4
	2	-x, y+1/2, -z+1/2	5.32	-79.5	-20.8	-30.8	93.6	-68.5
	2	x+1/2, -y+1/2, -z	8.25	-10.0	-1.0	-3.6	0.4	-14.1
	2	-x+1/2, -y, z+1/2	6.67	-11.5	-2.9	-12.4	5.8	-21.5
	2	-x, y+1/2, -z+1/2	8.39	-1.0	-0.8	-2.6	0.1	-3.9
2-CG	2	-x, y+1/2, -z+1/2	5.84	-8.6	-2.2	-19.7	16.0	-17.9
	2	x, y, z	6.84	-53.7	-11.4	-21.4	73.1	-38.7
	2	x+1/2, -y+1/2, -z	5.45	-66.0	-12.9	-27.0	78.9	-54.0
	2	-x, y+1/2, -z+1/2	7.45	-7.6	-1.8	-19.7	14.7	-17.5
	2	x, y, z	6.74	-54.3	-13.6	-18.0	67.3	-41.6
2-CM	1	-	4.33	-8.5	-2.6	-7.8	8.0	-12.8
	2	x, y, z	6.11	-84.2	-20.4	-28.4	107.1	-62.7
	2	-x, y+1/2, -z	6.34	-11.1	-2.9	-17.7	11.8	-22.1
	2	-x, y+1/2, -z	6.79	-35.8	-8.6	-16.9	41.6	-33.2
	2	-x, y+1/2, -z	6.48	-16.1	-2.8	-18.7	16.9	-24.9
	2	x, y, z	7.37	2.7	-1.0	-10.3	8.9	-1.4
	1	-	5.01	-47.0	-9.4	-6.6	54.2	-28.9
	1	-	4.69	-33.5	-6.7	-8.2	38.5	-23.7
	1	-	4.83	-57.7	-13.4	-5.5	69.7	-32.7
2-IG	2	x, y, z	6.35	-73.9	-14.8	-31.8	79.9	-67.5
	2	x+1/2, -y+1/2, -z	7.17	-9.0	-0.7	-12.6	20.1	-8.6
	2	x, y, z	6.67	-73.0	-18.1	-26.7	95.4	-54.9
	2	x+1/2, -y+1/2, -z	5.22	-9.5	-1.8	-25.3	25.4	-17.8
	2	-x, y+1/2, -z+1/2	6.99	-5.0	-1.5	-14.7	8.6	-13.9
	2	-x, y+1/2, -z+1/2	9.02	-5.0	-1.2	-20.2	16.3	-13.7
	2	x, y, z	9.21	-5.8	-0.7	-5.8	5.8	-8.2

Table S26. Enrichment ratios of atomic contacts calculated for significant (> 5% of HS) atomic contacts types in the 2-DG and its halogenated analogues

Compound	Interatomic contact	Enrichment ratio (E_{XY})
2-FG	H···H	0.83
	H···O	1.41
	H···F	1.27
2-FM	H···H	0.88
	H···O	1.33
	H···F	1.25
2-CG	H···H	0.81
	H···O	1.41

	H···Cl	1.35
2-IG	H···H	0.85
	H···O	1.38
	H···I	1.22
α-2-DG	H···H	0.92
	H···O	1.29
β-2-DG	H···H	0.92
	H···O	1.28

Table S27. Conformational analysis of the halogenated analogues of 2-DG in an aqueous solution and in the crystal state.

Comp.	Crystal state					Aqueous solution					
	Torsion angle		H-H distance		C5-C6 / O6-H6 rotamers	Torsion angle		H-H distance		C5-C6 (*gt*/gg/*tg* O6-H6 (*g*/*t*) [c] rotameric equilibria [%]	anomeric equilibrium (α/β) [%]
	angle	[°]	H-H	Å]		angle [b]	[°]	H-H	[Å]		
α-2-FG	-	-	-	-	-	H1-C1-C2-H2	+57	H1 H2	2.6	45/45/10	43/57
						H2-C2-C3-H3	+170	H3 H5	3.3	nd	
						H3-C3-C4-H4	-174				
						H4-C4-C5-H5	+180				
						H5-C5-C6-H6	nd				
						H5-C5-C6-H6′	nd				
						O5-C5-C6-O6 (ω)	-				
						C5-C6-O6-H06 (θ)	-				
β-2-FG	H1-C1-C2-H2	+172.4	H1 H3	2.70	*gg*/*trans*	H1-C1-C2-H2	+174	H1 H3	2.4	50/40/10	
	H2-C2-C3-H3	-167.2	H1 H5	2.35		H2-C2-C3-H3	+171	H1 H5	2.2	nd	
	H3-C3-C4-H4	+168.6	H3 H5	2.63		H3-C3-C4-H4	+177	H3 H5	3.1		
	H4-C4-C5-H5	-177.0	H4 H6	3.60		H4-C4-C5-H5	+173	H4 H6	3.9		
	H5-C5-C6-H6	+60.7	H5 H6	2.35		H5-C5-C6-H6	nd	H5 H6	2.7		
	H5-C5-C6-H6′	-58.2	H5 H6′	2.34		H5-C5-C6-H6′	nd	H5 H6′	3.5		
	O5-C5-C6-O6 (*ω*)	-61.0	H6 H6′	1.57		O5-C5-C6-O6 (ω)	-	H6 H6′	1.8		
	C5-C6-O6-H06 (*θ*)	+157.7				C5-C6-O6-H06 (θ)	-				
α-2-FM	-					H1-C1-C2-H2	-70	H1 H2	2.4	50/45/5	65/35
						H2-C2-C3-H3	-66	H2 H3	2.4	nd	
						H3-C3-C4-H4	+180				
						H4-C4-C5-H5	+174				
						H5-C5-C6-H6	nd				
						H5-C5-C6-H6′	nd				
						O5-C5-C6-O6 (ω)	-				
						C5-C6-O6-H06 (θ)	-				

β-2-FM	H1-C1-C2-H2	+54.6	H1 H2	2.34	*gg*/*trans*	H1-C1-C2-H2	nd	H1 H2	2.8	60/30/10	
	H2-C2-C3-H3	-53.1	H1 H3	2.69		H2-C2-C3-H3	-61	H1 H3	2.7		
	H3-C3-C4-H4	+173.2	H1 H5	2.40		H3-C3-C4-H4	-174	H1 H5	2.5		
	H4-C4-C5-H5	-179.7	H2 H3	2.33		H4-C4-C5-H5	-179	H2 H3	2.8		
	H5-C5-C6-H6	+57.4	H3 H5	2.57		H5-C5-C6-H6	nd	H3 H5	2.8		
	H5-C5-C6-H6′	-61.5	H5 H6	2.33		H5-C5-C6-H6′	nd	H5 H6	2.5		
	O5-C5-C6-O6 (ω)	-64.2	H5 H6′	2.35		O5-C5-C6-O6 (ω)	-	H5 H6′	2.4		
	C5-C6-O6-H06 (θ)	-170.4	H6 H6′	1.57		C5-C6-O6-H06 (θ)	-	H6 H6′	1.8		
α-2,2′-FG						H3-C3-C4-H4	+180	H3 H5	2.1	50/45/5	68/32
						H4-C4-C5-H5	-179	H4 H6	2.7	nd	
	-					H5-C5-C6-H6	-	H4 H6′	2.2		
						H5-C5-C6-H6′	-	H5 H6	2.5		
β-2,2′-FG						H3-C3-C4-H4	+170	H1 H3	2.4	nd	
								H1 H5	2.2		
	-							H6 H6′	1.8		
α-2-CG						H1-C1-C2-H2	+61	H1 H2	2.5	45/45/10	36/64
						H2-C2-C3-H3	+175	H1 H5	3.3	75/25	
						H3-C3-C4-H4	-175	H2 H4	2.7		
						H4-C4-C5-H5	-179	H3 H5	3.1		
						H5-C5-C6-H6	nd				
						H5-C5-C6-H6′	nd				
						O5-C5-C6-O6 (ω)	-				
						C5-C6-O6-H06 (θ)	-				
β-2-CG	H1-C1-C2-H2	+176.9	H1 H3	2.64	*gg*/*gauche*	H1-C1-C2-H2	+179	H1 H3	2.5	60/30/10	
	H2-C2-C3-H3	-169.3	H1 H5	2.22		H2-C2-C3-H3	+168	H1 H5	2.2	80/20	
	H3-C3-C4-H4	+170.8	H2 H4	2.67		H3-C3-C4-H4	-175	H2 H4	2.5		
	H4-C4-C5-H5	+178.4	H3 H5	2.54		H4-C4-C5-H5	-179	H3 H5	2.4		
	H5-C5-C6-H6	+55.9	H4 H6	3.09		H5-C5-C6-H6	nd	H4 H6	3.1		
	H5-C5-C6-H6′	-60.7	H5 H6	2.30		H5-C5-C6-H6′	nd	H5 H6	2.4		
	O5-C5-C6-O6 (ω)	-64.3	H5 H6′	2.33		O5-C5-C6-O6 (ω)	-	H5 H6′	2.5		
	C5-C6-O6-H06 (θ)	-68.7	H6 H6′	1.57		C5-C6-O6-H06 (θ)	-	H6 H6′	1.8		
α-2-CM						H1-C1-C2-H2	+73	H1 H2	2.4	50/40/10	46/54
						H2-C2-C3-H3	-57	H2 H3	2.3	nd	
						H3-C3-C4-H4	-175	H3 H5	2.4		
	-					H4-C4-C5-H5	+180				
						H5-C5-C6-H6	nd				

						H5-C5-C6-H6′	nd				
						O5-C5-C6-O6 (ω)	-				
						C5-C6-O6-H06 (θ)	-				
β-2-CM	H1-C1-C2-H2	+54.9	H1 H2	2.33	*gt*/[a]	H1-C1-C2-H2	+72	H1 H2	2.3	60/30/10	
	H2-C2-C3-H3	-51.2	H1 H3	2.60		H2-C2-C3-H3	-58	H1 H3	2.3	nd	
	H3-C3-C4-H4	172.0	H1 H5	2.33		H3-C3-C4-H4	-175	H1 H5	2.2		
	H4-C4-C5-H5	-176.6	H2 H3	2.30		H4-C4-C5-H5	-177	H2 H3	2.3		
	H5-C5-C6-H6	-174.7	H3 H5	2.57		H5-C5-C6-H6	nd	H3 H5	2.5		
	H5-C5-C6-H6′	+67.5	H5 H6	2.39		H5-C5-C6-H6′	nd	H5 H6	2.6		
	O5-C5-C6-O6 (ω)	+65.6	H5 H6′	2.83		O5-C5-C6-O6 (ω)	-	H5 H6′	3.0		
	C5-C6-O6-H06 (θ)	-117.8	H6 H6′	1.57		C5-C6-O6-H06 (θ)	-	H6 H6′	1.8		
α-2-IG	H1-C1-C2-H2	+52.0	H1 H2	2.28	*gg*/[a]	H1-C1-C2-H2	+62	H1 H2	2.5	45/45/10	34/66
	H2-C2-C3-H3	-174.9	H2 H4	2.47		H2-C2-C3-H3	+180	H2 H4	2.8	nd	
	H3-C3-C4-H4	+176.9	H6 H6′	1.57		H3-C3-C4-H4	-176	H6 H6′	1.8		
	H4-C4-C5-H5	+175.7				H4-C4-C5-H5	-179				
	H5-C5-C6-H6	+51.6				H5-C5-C6-H6	nd				
	H5-C5-C6-H6′	-65.7				H5-C5-C6-H6′	nd				
	O5-C5-C6-O6 (ω)	-69.2				O5-C5-C6-O6 (ω)	-				
	C5-C6-O6-H06 (θ)	111.4				C5-C6-O6-H06 (θ)	-				
β-2-IG	-					H1-C1-C2-H2	+179	H1 H3	2.4	60/30/10	
						H2-C2-C3-H3	+177	H1	2.2	nd	
						H3-C3-C4-H4	-176	H45	2.6		
						H4-C4-C5-H5	+180	H5 H6			
						H5-C5-C6-H6	nd				
						H5-C5-C6-H6′	nd				
						O5-C5-C6-O6 (ω)	-				
						C5-C6-O6-H06 (θ)	-				
α-2-IM	-					H1-C1-C2-H2	-75	H1 H2	2.6	50/40/10	38/62
						H2-C2-C3-H3	-56	H2 H3	2.5	90/10	
						H3-C3-C4-H4	-176	H3 H5	2.6		
						H4-C4-C5-H5	-179	H5 H6	2.8		
						H5-C5-C6-H6	nd	H5 H6′	3.0		
						H5-C5-C6-H6′	nd				
						O5-C5-C6-O6 (ω)	-				
						C5-C6-O6-H06 (θ)	-				
β-2-IM	-					H1-C1-C2-H2	nd	H1 H2	2.3	60/30/10	
						H2-C2-C3-H3	+52	H1 H3	2.3	nd	

						H3-C3-C4-H4	-175	H1 H5	2.2		
						H4-C4-C5-H5	-179	H2 H3	2.3		
						H5-C5-C6-H6	nd	H3 H5	2.6		
						H5-C5-C6-H6′	nd	H5 H6	2.5		
						O5-C5-C6-O6 (ω)	-	H5 H6′	2.9		
						C5-C6-O6-H06 (θ)	-	H6 H6′	1.8		
α-2-DG	H1-C1-C2-H2	-68.7	H1 H2	2.41	*gt/*[a]	H1-C1-C2-H2	+58	H1 H2	2.5	45/45/10	49/51
	H1-C1-C2-H2′	49.0	H1 H2′	2.30		H1-C1-C2-H2′	nd	H1 H2′	2.5	nd	
	H2-C2-C3-H3	-52.9	H2 H2′	1.60		H2-C2-C3-H3	-56	H2 H2′	1.8		
	H2′-C2-C3-H3	-170.6	H2 H3	2.33		H2′-C2-C3-H3	+180	H2 H3	2.5		
	H3-C3-C4-H4	+175.0	H2′ H4	2.61		H3-C3-C4-H4	-175	H2′ H4	2.6		
	H4-C4-C5-H5	-178.6	H3 H5	2.53		H4-C4-C5-H5	-179	H3 H5	2.5		
	H5-C5-C6-H6	+85.0	H6 H6′	1.60		H5-C5-C6-H6	nd	H6 H6′	1.8		
	H5-C5-C6-H6′	-157.1				H5-C5-C6-H6′	nd				
	O5-C5-C6-O6 (*ω*)	81.9				O5-C5-C6-O6 (ω)	-				
	C5-C6-O6-H06 (*θ*)	-119.6				C5-C6-O6-H06 (θ)	-				
β-2-DG	H1-C1-C2-H2	+54.8	H1 H2	2.43	*gg/trans*						
	H1-C1-C2-H2′	+174.9	H1 H2′	3.03		H1-C1-C2-H2	+59	H1 H2	2.4	60/30/10	
	H2-C2-C3-H3	-51.1	H1 H3	2.65		H1-C1-C2-H2′	+179	H1 H2′	3.4	nd	
	H2′-C2-C3-H3	-170.7	H1 H5	2.39		H2-C2-C3-H3	-56	H1 H3	2.4		
	H3-C3-C4-H4	+170.6	H2 H2′	1.76		H2′-C2-C3-H3	+180	H1 H5	2.2		
	H4-C4-C5-H5	-177.1	H2 H3	2.39		H3-C3-C4-H4	-175	H2 H2′	1.8		
	H5-C5-C6-H6	+58.0	H2′ H4	2.66		H4-C4-C5-H5	-179	H2 H3	2.4		
	H5-C5-C6-H6′	-61.8	H4 H6	3.12		H5-C5-C6-H6	nd	H2′ H4	2.5		
	O5-C5-C6-O6 (*ω*)	-64.8	H5 H6	2.42		H5-C5-C6-H6′	nd	H4 H6	2.5		
	C5-C6-O6-H06 (*θ*)	157.4	H5 H6′	2.45		O5-C5-C6-O6 (ω)	-	H5 H6	2.2		
			H6 H6′	1.77		C5-C6-O6-H06 (θ)	-	H5 H6′	2.5		
								H6 H6′	1.8		

[a] conformation between *cis* and *trans* conformers

[b] for torsion angles H5-C5-C6-H6 and H5-C5-C6-H6′ the values could not be determined for the each rotamer using available NMR data. In the case of ω angle it has been assumed that values are -60° (±5), -60° (±5), -180° (±5) for the rotamers *gg, gt, tg* respectively. The value of θ was assumed to be (±) 60° or 180 ° for *cis* andand *gauche* conformers respectively

[c] *estimated only on the basis of* $J_{6,6'}$ *values*

Table S28. ^{1}H-^{13}C HSQC and ^{1}H NMR data for halogenated analogues of 2-DG and the parental compound. NMR shifts values are measured in ppm and *J*-coupling constants in Hz

Compound	H1; C1	H2; C2	H3; C3	H4; C4	H5; C5	H6; H6′; C6
	$J_{1,2}$	$J_{2,3}$	$J_{3,4}$	$J_{4,5}$	$J_{5,6R}$; $J_{5,6S}$	$J_{6,6}$
α-2-FG	5.31; 91.65	4.28; 92.22	3.96; 73.17	3.46; 71.22	3.85; 73.27	-

	3.9	9.5	9.6	10.0	5.3; 2.2	
β-2-FG	4.77; 95.53	3.97; 94.89	3.78; 76.04	3.22; 71.22	3.26; 78.06	-
	7.9	9.1	9.5	10.0	5.9; 2.2	
α-2-FM	5.24; 93.32	4.63; 92.38	3.79; 71.47	3.46; 68.87	3.85; 74.32	-
	2.2	2.3	10.0	10.3	5.7; 2.1	-
β-2-FM	4.87; 94.36	4.67; 93.35	3.76; 73.86	3.38; 68.65	3.20; 78.13	-
	nd	2.6	10.0	9.7	6.7; 2.3	-
α-2-diFG	5.14; 92.69	-;	4.03; 72.41	3.61; 70.42	3.94; 73.83	-
	-		10.1	10.4	5.5; 2.0	
β-2-diFG	4.89; 93.60	-;	3.67; 74.89			
	-		8.6	nd	nd; nd	-
α-2-CG	5.22; 94.09		3.70; 74.82	3.35; 72.39	3.91; 73.70	-
	3.4	10.4	9.5	10.0	5.2; 2.4	11.8
β-2-CG	4.72; 97.85	3.33; 65.07	3.65; 78.22	3.19; 72.07	3.52; 78.03	-
	8.5	8.7	9.5	9.5	6.4; 2.6	12.0
α-2-CM	5.25; 95.92	4,24; 64.02	4.02; 70.92	3.52; 68.50	3.88; 74.95	-
	1.6	3.8	9.6	10.2	5.8; 2.3	-
β-2-CM	5.02; 94.03	4.34; 66.96	3.83; 73.84	3.66; 68.16	3.44; 78.75	-
	1.2	3.5	9.6	9.8	6.5; 2.4	-
α-2-IG	5.30; 95.36	4.01; 34.91	3.64; 75.32	3.33; 72.57	3.93; 74.00	-
	3.2	11.2	9.3	9.9	5.2; 2.4	-
β-2-IG	4.88; 98.61	3.78; 38.42	3.47; 79.08	3.16; 72.26	3.51; 77.97	-
	9.2	10.6	9.3	9.9	6.0; 2.0	-
α-2-IM	5.47; 97.49	4.38; 39.08	3.17; 69.85	3.48; 70.95	3.92; 75.28	-
	1.4	4.2	9.3	9.7	5.9; 2.3	12.3
β-2-IM	4.11; 93.44	4.50; 46.48	3.10; 72.89	3.53; 70.66	3.47; 78.96	-
	1.5	3.9	9.4	9.7	6.4; 2.2	-
α-2-DG	5.25; 93.27		3.93; 69.94	3.23; 73.14	3.80; 73.98	-

	nd; 3.5	2.00; 1.68;	9.4	9.7	5.3; 2.5	-
		39.24				
β-2-DG	4.95; 95.37	13.4[b]; 12.4;	3.71; 72.44	3.03; 72.82	3.37; 77.98	-
	10.1; 1.8	5.1	9.4	9.6	6.6; 2.6	-
		2.24; 1.49;				
		41.47				
		12.4[b]; 12.0;				
		5.1				

[a] Taken from Fokt 2009

[b] $J_{2,2'}$

Table S29. ^{19}F NMR data for fluorinated analogues of 2-DG. NMR shifts values are measured in ppm and *J*-coupling constants in Hz

Compound	$J_{F2,H1}$	F2; $J_{F2,H2}$	$J_{F2,H3}$	$J_{F2,H4}$	$J_{F2,H5}$	$J_{F2,H6}$
α-2-FG	0.0	;49.4	13.4	3.3	nd	nd
β-2-FG	2.4	51.5	15.1	4.4	nd	nd
α-2-FM	7.3	49.2	31.3	nd	nd	nd
β-2-FM	20.4	51.5	30.9	nd	nd	nd
α-2-diFG	0.1; 6.5	; -	22.4; 5.1	nd	nd	nd
β-2-diFG	0.7; 16.0	; -	21.5; 5.8	nd	nd	nd

Table S30. The geometry of hydrogen bonds between 2-FG and protein residues for 3K4L structure

D	A	D–H (Å)	d(D···A) (Å)
SHG 901 O1	ASP 452 OD2	1.863	2.637
SHG 901 O3	HIS 548 NE2	1.759	2.518
SHG 901 O4	VAL 546 O	1.759	2.787
GLN 448 HE22	SHG 901 F1	2.135	2.960
ASN 593 ND2	SHG 901 O3	1.884	2.692

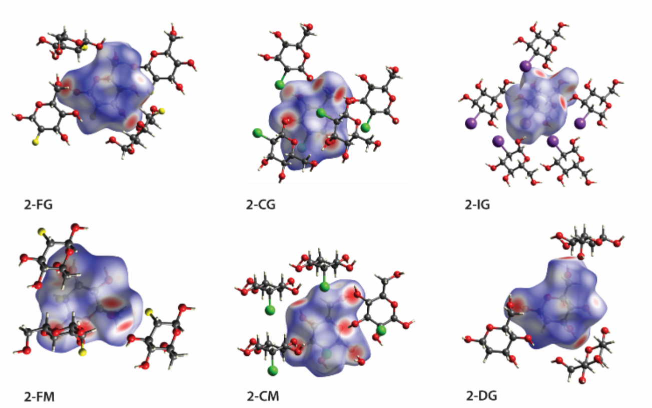

Figure S1. Hirshfeld surfaces mapped onto d_e. Intermolecular contacts which are closer than the sum of their Van der Waals radii are highlighted in red on the de surface, whereas longer contacts are blue. Contacts having similar lengths to the radii sum are depicted as white.

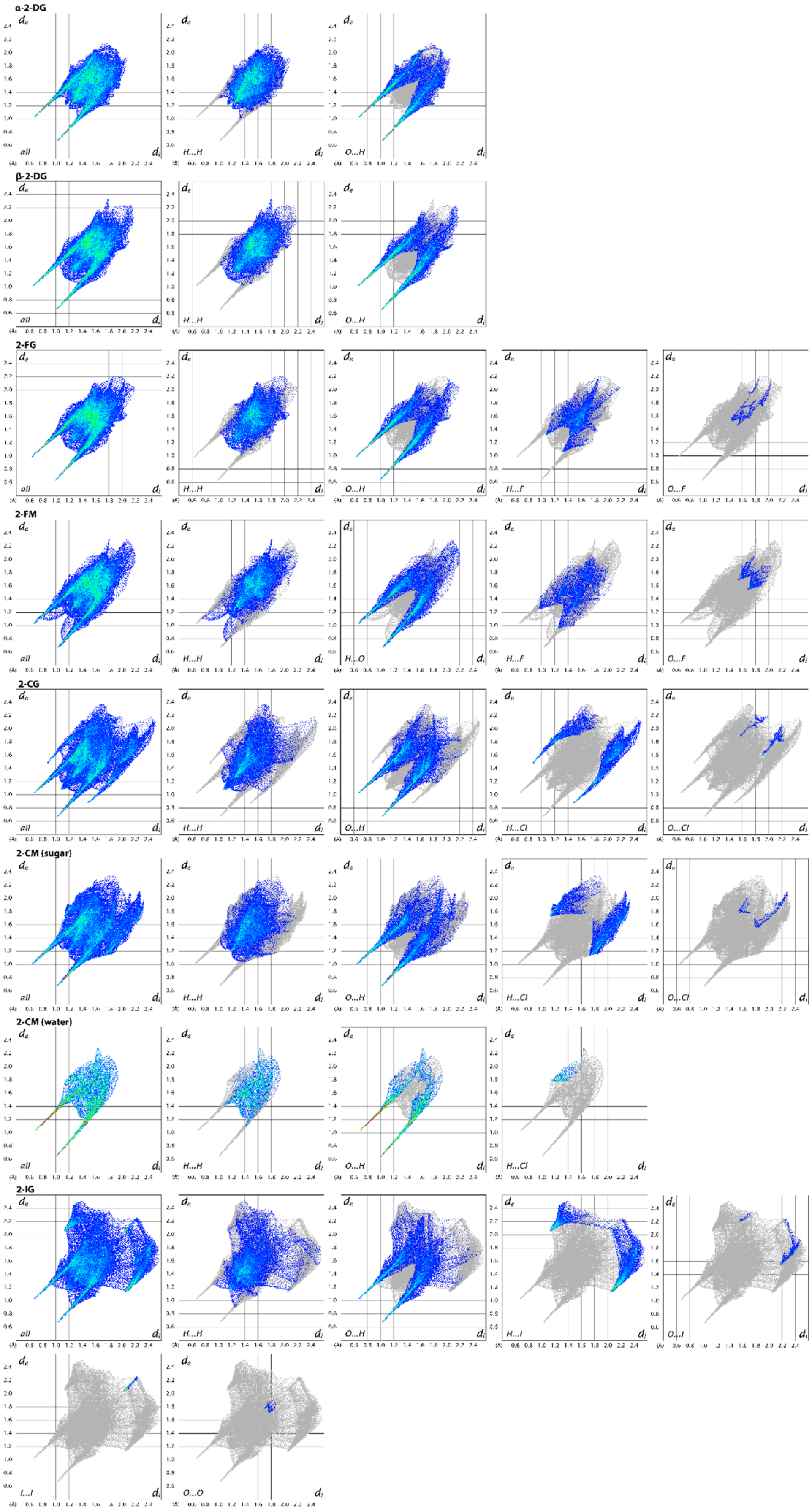

Figure 2. Contribution of different X-X contacts for each crystal lattice discussed in the article.

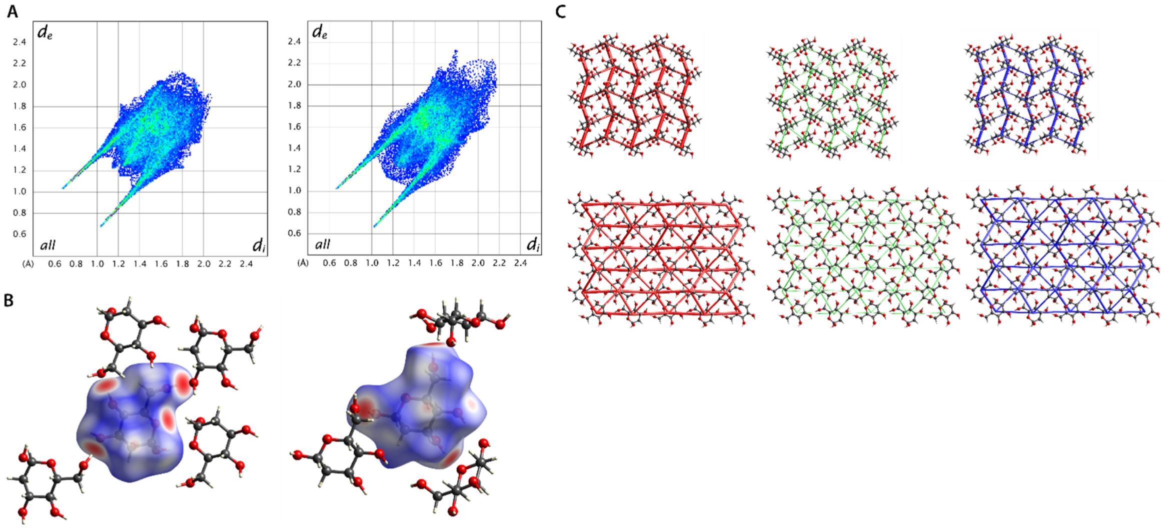

Figure S3. A. comparison between fingerprint plots between α (left) and β (right) anomers of 2-DG **B**. Comparison between Hirshfeld surfaces between these structures (α on the left and β on the right) and their energy frameworks (**C**) where β anomer is on the top and α in the bottom.

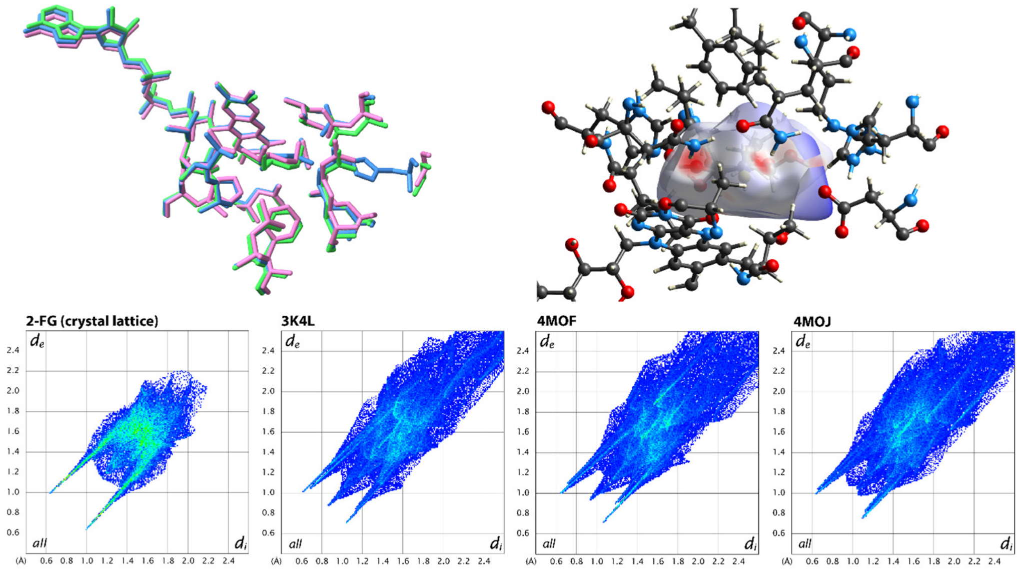

Figure S4. Structural alignment of amino-acid residues involving in 2-FG binding in 3K4L (green), 4MOF (blue) and 4 MOJ (pink, top-right) an exemplary Hirshfeld surface calculated for 2-FG in 3K4L complex (top-left) and comparison between fingerprint plots for 2-FG in crystal lattice and different protein-ligand complexes.

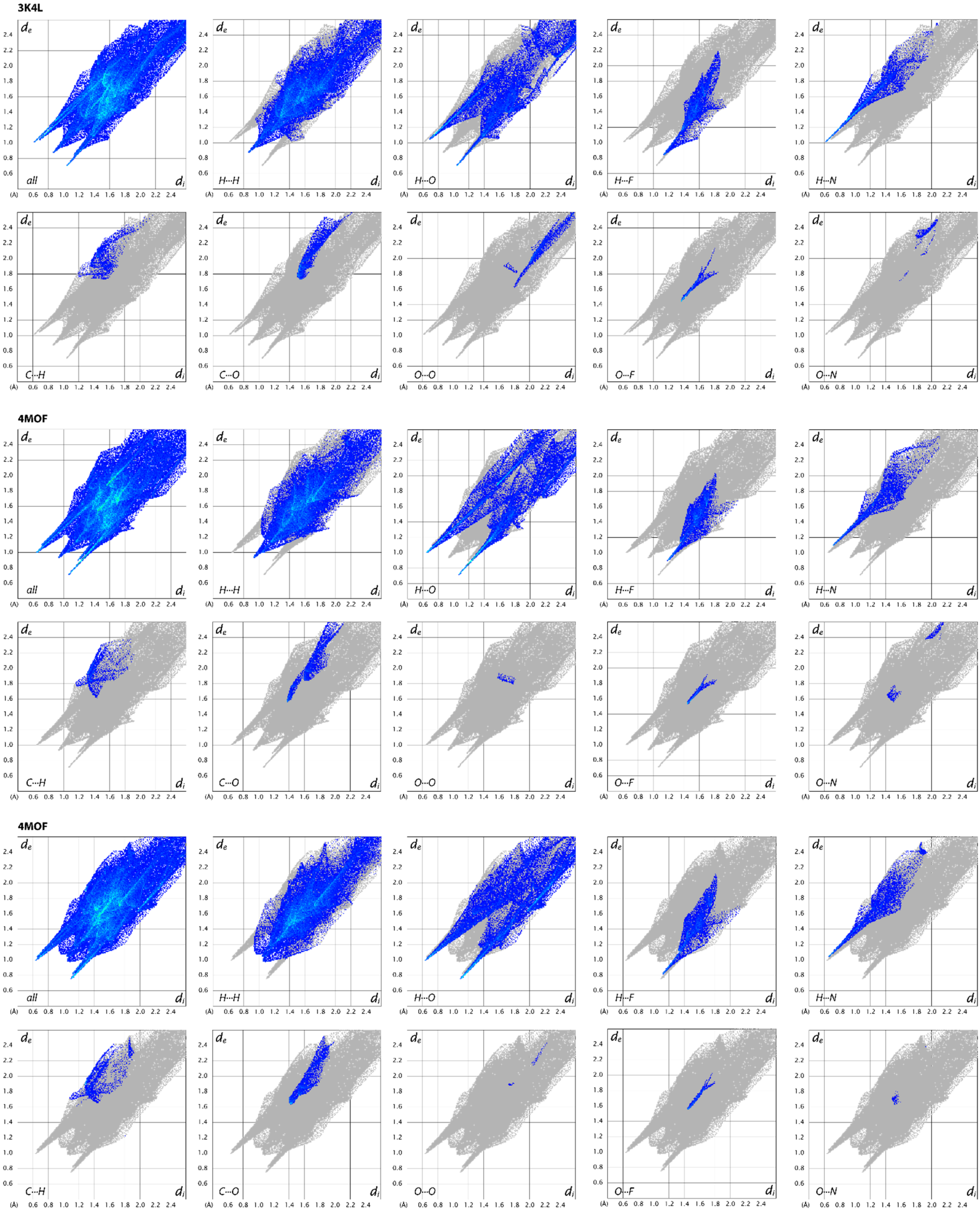

Figure S5. Contribution of different X-X contacts for each protein-ligand complex discussed in the article
